# Variant Impact Predictor database (VIPdb), version 2: Trends from 25 years of genetic variant impact predictors

**DOI:** 10.1101/2024.06.25.600283

**Published:** 2024-06-28

**Authors:** Yu-Jen Lin, Arul S. Menon, Zhiqiang Hu, Steven E. Brenner

**Affiliations:** 1 Department of Molecular and Cell Biology, University of California, Berkeley, California 94720, USA; 2 Center for Computational Biology, University of California, Berkeley, California 94720, USA; 3 College of Computing, Data Science, and Society, University of California, Berkeley, California 94720, USA; 4 Department of Plant and Microbial Biology, University of California, Berkeley, California 94720, USA; 5 Currently at: Illumina, Foster City, California 94404, USA

**Keywords:** VIPdb, variant impact predictor (VIP), variant effect predictor (VEP), genomic variant, variant interpretation, SNV, SV, indel, genotype-phenotype relationship

## Abstract

**Background:**

Variant interpretation is essential for identifying patients’ disease-causing genetic variants amongst the millions detected in their genomes. Hundreds of Variant Impact Predictors (VIPs), also known as Variant Effect Predictors (VEPs), have been developed for this purpose, with a variety of methodologies and goals. To facilitate the exploration of available VIP options, we have created the Variant Impact Predictor database (VIPdb).

**Results:**

The Variant Impact Predictor database (VIPdb) version 2 presents a collection of VIPs developed over the past 25 years, summarizing their characteristics, ClinGen calibrated scores, CAGI assessment results, publication details, access information, and citation patterns. We previously summarized 217 VIPs and their features in VIPdb in 2019. Building upon this foundation, we identified and categorized an additional 186 VIPs, resulting in a total of 403 VIPs in VIPdb version 2. The majority of the VIPs have the capacity to predict the impacts of single nucleotide variants and nonsynonymous variants. More VIPs tailored to predict the impacts of insertions and deletions have been developed since the 2010s. In contrast, relatively few VIPs are dedicated to the prediction of splicing, structural, synonymous, and regulatory variants. The increasing rate of citations to VIPs reflects the ongoing growth in their use, and the evolving trends in citations reveal development in the field and individual methods.

**Conclusions:**

VIPdb version 2 summarizes 403 VIPs and their features, potentially facilitating VIP exploration for various variant interpretation applications.

**Availability:**

VIPdb version 2 is available at https://genomeinterpretation.org/vipdb

## Background

Advances in sequencing technologies, including gene panels, whole exome sequencing, whole genome sequencing, and long read sequencing, have revolutionized the investigation of genetic variation on a large scale and hence have accelerated the discovery of novel genetic etiologies of diseases and improved the efficiency of diagnosis (1, 2). Typically, thousands to millions of variants are identified in each individual (3, 4), making it challenging to distinguish disease-causing variants from non-contributory ones. Consequently, methods to predict the impacts of variants being disease-causing are essential (5, 6).

This need prompted the development of Variant Impact Predictors (VIPs), tools or databases designed to predict the consequences of genetic variants. Hundreds of genetic VIPs have been developed, with a variety of methodologies and goals (7). Some overlapping categories of variants considered by different tools are single nucleotide variations (SNVs), insertions and deletions (indels), structural variations (SVs), nonsynonymous variants, synonymous variants, splicing variants, and regulatory variants. VIPs are designed for different contexts, such as for germline variants, somatic variants, or specific diseases or genes. The variety of VIPs underscores the complex nature of variant interpretation and poses a challenge for users in identifying the most suitable VIPs for their specific needs.

Many computational impact prediction methods have been developed, yet the field lacks a clear consensus on their appropriate use and interpretation (8). Recognizing the need for an organized approach to explore available VIPs, several research entities have constructed resources facilitating the informed use of VIPs. Initiatives like the Critical Assessment of Genome Interpretation (CAGI) conduct community experiments to assess VIPs across different variant types and contexts (8, 9, 10). The dbNSFP (database for Nonsynonymous Single-nucleotide polymorphisms’ Functional Predictions) hosts precomputes of several VIP results (11). OpenCRAVAT integrates hundreds of VIP analyses of cancer-related variants in one platform, enhancing accessibility for users (12). These resources have played an important role in introducing users to VIP options. Consequently, we developed VIPdb to serve as a comprehensive resource for exploring VIPs.

To systematically evaluate the pathogenicity of a variant in a clinical laboratory, ACMG/AMP has established guidelines for interpreting genetic variants that integrate several lines of evidence, including population data, functional data, segregation data, and computational prediction (13). Historically, VIPs provided only supporting evidence in determining the pathogenicity or benignity of variants in clinical settings. However, recent ClinGen clinical recommendations allow VIPs the potential to provide stronger evidence (14). This greater role for VIPs in providing evidence for clinical decisions could improve genetic disease diagnosis.

The Variant Impact Predictor database (VIPdb) offers a curation of available computational tools for predicting variant impact. Initially established in 2007 and 2010 (15), the database was last updated in 2019 (7). VIPdb version 2 is a comprehensive update through January 2, 2024.

## Implementation

Our identification of VIPs involved searching for potential VIPs and examining their articles to determine whether they should be included in VIPdb. In the initial step, we searched the literature using the query “(((tool(Title]) OR (pipeline(Title])) AND (variant(Title/Abstract]))” on PubMed and collected potential VIPs citing pioneering VIPs (SIFT, PolyPhen, ANNOVAR, and SnpEff) (16, 17, 18, 19, 20, 21, 22, 23, 24, 25, 26, 27). Additionally, we gathered potential VIPs from existing databases such as OpenCRAVAT and dbNSFP, as well as from submissions by VIP developers. Subsequently, we examined the literature and included only programs capable of handling variant data, such as VCF files, rsID, or location in the genome, and providing evidence or predictions of the variant impacts. Overall, this resulted in the identification of 186 additional VIPs, augmenting the VIPdb to a total of 403 VIPs (16, 17, 19, 21, 22, 23, 24, 25, 26, 27, 28, 29, 30, 31, 32, 33, 34, 35, 36, 37, 38, 39, 40, 41, 42, 43, 44, 45, 46, 47, 48, 49, 50, 51, 52, 53, 54, 55, 56, 57, 58, 59, 60, 61, 62, 63, 64, 65, 66, 67, 68, 69, 70, 71, 72, 73, 74, 75, 76, 77, 78, 79, 80, 81, 82, 83, 84, 85, 86, 87, 88, 89, 90, 91, 92, 93, 94, 95, 96, 97, 98, 99, 100, 101, 102, 103, 104, 105, 106, 107, 108, 109, 110, 111, 112, 113, 114, 115, 116, 117, 118, 119, 120, 121, 122, 123, 124, 125, 126, 127, 128, 129, 130, 131, 132, 133, 134, 135, 136, 137, 138, 139, 140, 141, 142, 143, 144, 145, 146, 147, 148, 149, 150, 151, 152, 153, 154, 155, 156, 157, 158, 159, 160, 161, 162, 163, 164, 165, 166, 167, 168, 169, 170, 171, 172, 173, 174, 175, 176, 177, 178, 179, 180, 181, 182, 183, 184, 185, 186, 187, 188, 189, 190, 191, 192, 193, 194, 195, 196, 197, 198, 199, 200, 201, 202, 203, 204, 205, 206, 207, 208, 209, 210, 211, 212, 213, 214, 215, 216, 217, 218, 219, 220, 221, 222, 223, 224, 225, 226, 227, 228, 229, 230, 231, 232, 233) (11, 18, 20, 234, 235, 236, 237, 238, 239, 240, 241, 242, 243, 244, 245, 246, 247, 248, 249, 250, 251, 252, 253, 254, 255, 256, 257, 258, 259, 260, 261, 262, 263, 264, 265, 266, 267, 268, 269, 270, 271, 272, 273, 274, 275, 276, 277, 278, 279, 280, 281, 282, 283, 284, 285, 286, 287, 288, 289, 290, 291, 292, 293, 294, 295, 296, 297, 298, 299, 300, 301, 302, 303, 304, 305, 306, 307, 308, 309, 310, 311, 312, 313, 314, 315, 316, 317, 318, 319, 320, 321, 322, 323, 324, 325, 326, 327, 328, 329, 330, 331, 332, 333, 334, 335, 336, 337, 338, 339, 340, 341, 342, 343, 344, 345, 346, 347, 348, 349, 350, 351, 352, 353, 354, 355, 356, 357, 358, 359, 360, 361, 362, 363, 364, 365, 366, 367, 368, 369, 370, 371, 372, 373, 374, 375, 376, 377, 378, 379, 380, 381, 382, 383, 384, 385, 386, 387, 388, 389, 390, 391, 392, 393, 394, 395, 396, 397, 398, 399, 400, 401, 402, 403, 404, 405, 406, 407, 408, 409, 410, 411, 412, 413, 414, 415).

To facilitate users’ exploration of available VIPs, we described key features of each VIP. VIPs primarily designed for variant impact prediction were labeled as such. VIPs not originally designed for variant impact prediction but nonetheless used for this purpose, such as those estimating conservation scores and population allele frequencies, were categorized as non-primary. VIPs containing clinical classifications, functional data, or population data were categorized as databases, whereas VIPs utilizing databases for computing variant impact predictions were classified as non-databases. Furthermore, as VIPs are designed for different types of genetic variants, we classified the VIPs according to the following overlapping categories of input: single nucleotide variant (SNV), insertion and deletion (indel) variant, structural variant (SV), nonsynonymous/nonsense variant, synonymous variant, splicing variant, and regulatory region variants, with some overlap among these categories. Licensing information, including whether the VIP is free for academic or commercial use, was also included. In addition, we provided details about accessing VIPs, such as homepage links and source code availability.

In VIPdb version 2, we have made enhancements to inform clinical decision-making. We incorporated calibrated threshold scores recommended by ClinGen for clinical use (14) with ACMG/AMP guidelines for variant classification (13). Additionally, we included community assessment results from the CAGI 6 Annotate All Missense / Missense Marathon challenge (416) to enable users to compare the overall performance of methods and the performance on subsets with high specificity or high sensitivity.

To understand the trends of genetic VIPs over 25 years, we conducted a citation analysis. We utilized the Entrez module in Biopython to retrieve citation information from the PubMed database. Specifically, the elink function was employed to collect the number of articles citing each VIP, and the esummary function allowed for the collection of publication years for these citations. These functions facilitated the automatic collection of citation numbers by year for each VIP.

In summary, VIPdb version 2 presents a collection of 403 VIPs developed over the last 25 years, with their characteristics, citation patterns, publication details, and access information. VIPdb version 2 is publicly accessible at https://genomeinterpretation.org/vipdb

## Results

We incorporated 186 additional VIPs into VIPdb version 2, alongside the existing 217 VIPs in the previous version of VIPdb. We summarized the characteristics of the 403 VIPs in VIPdb version 2. Among the 403 VIPs in VIPdb version 2, 274 are core VIPs, defined as VIPs primarily designed for variant impact prediction and not a database.

**Figure. 1.**
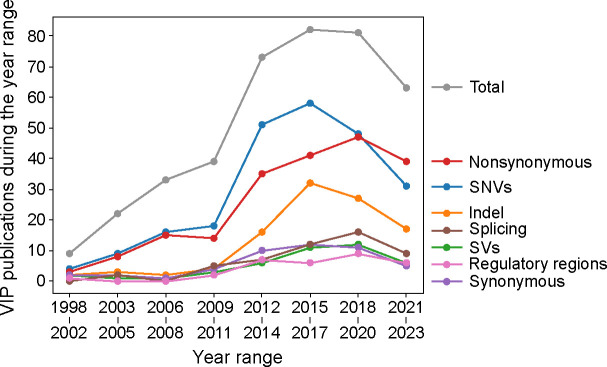
VIP variant type focus.

An analysis of the variant type used by VIP showed a predominant focus on predicting the impacts of single nucleotide variants (SNVs) and nonsynonymous variants (Fig. 1). Since the 2010s, there has been a notable surge in the development of VIPs tailored for insertions and deletions (indels), while VIPs dedicated to predicting the impacts of splicing, structural, synonymous, and regulatory variants have grown more modestly (Fig. 1). These observations about VIP variant type not only highlight current focus on but also identify areas that have been less explored, suggesting potential directions for future research.

**Figure 2.**
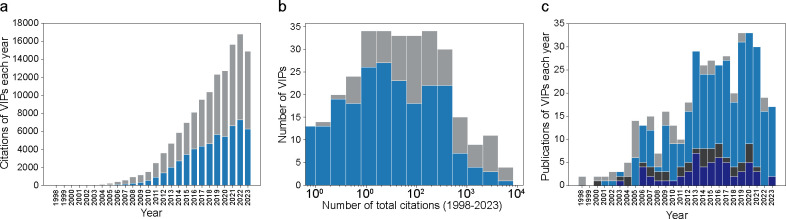
Citation and publication analysis of 403 VIPs. (a) Citations each year for 274 core VIPs (blue) and 126 non-core VIPs (gray). (b) Histogram of total citations for core VIPs (blue) and non-core VIPs (gray). (c) VIPs published per year, with original publications in light blue (core) and light gray (non-core), and subsequent publications in dark blue (core) and dark gray (non-core).

The citation rate of VIPs continues to rise, while the annual publications of VIPs have reached a plateau (Fig. 2). The increasing citation rates for both the 274 core VIPs and the 129 non-core VIPs reflect the ongoing growth of VIP usage (Fig. 2A). The median total citation for VIPs is 41 from 1998 to 2023, with a 95% quantile of 2612 citations (Fig. 2B). Annual publication showed a stabilization in VIP publications, with some being subsequent publications from previous work (Fig. 2C).

**Figure 3.**
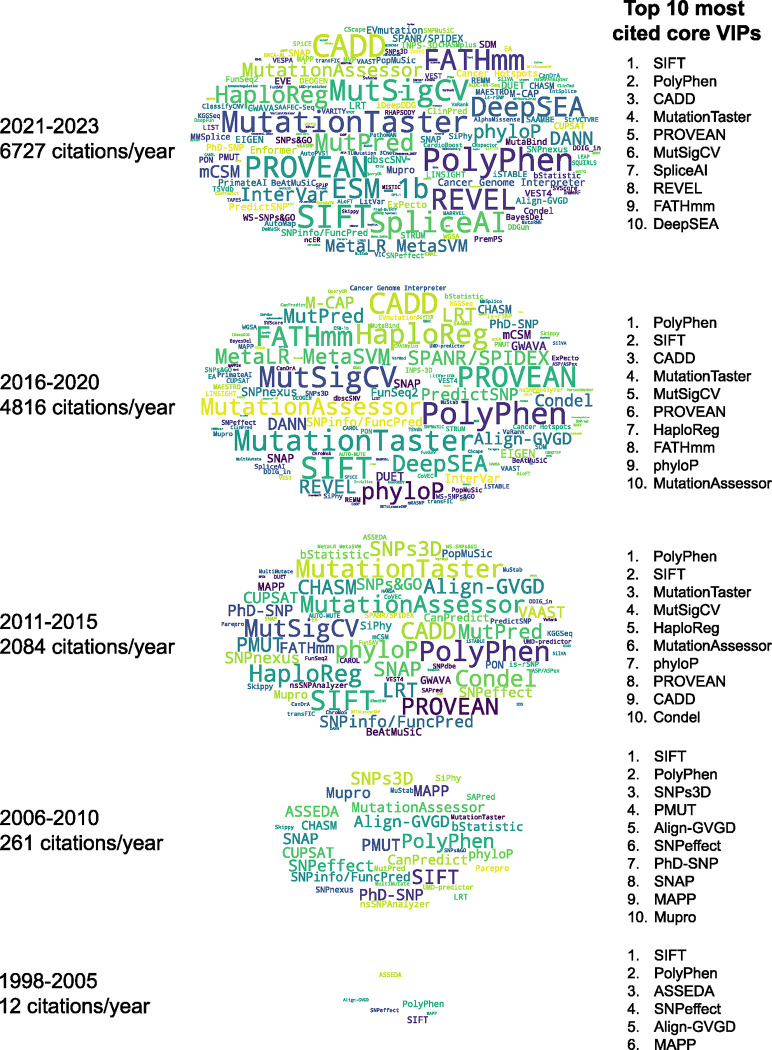
Citation trend of 274 core VIPs (1998 to 2023). Word clouds representing core VIPs over a specific time period, using cumulative citations for core VIPs with multiple publications. Font sizes in the word clouds correspond to the logarithm of citation counts for each period, and cloud heights are scaled by the logarithm of the annual citation averages. The top 10 most cited core VIPs during the period are listed. Note: Core VIPs are methods primarily designed for variant impact prediction and are not classified as databases.

**Figure 4.**
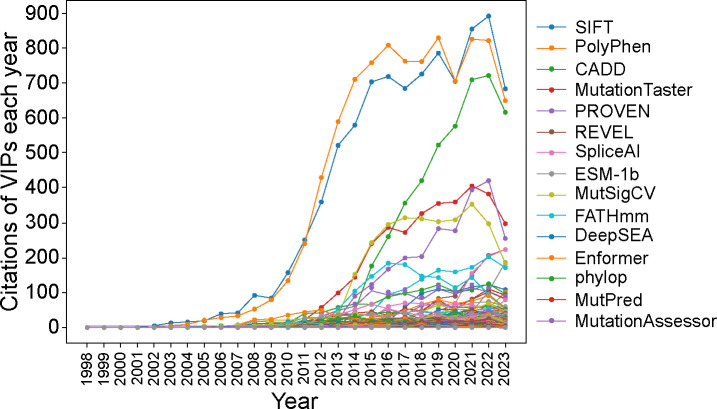
Citation trend of the top 15 most cited core VIPs in the year 2023. Note: Core VIPs are methods primarily designed for variant impact prediction and are not classified as databases.

The citation trend of 274 core VIPs from 1998 to 2023 is shown in Fig. 3 and 4. The citation analysis revealed that SIFT and PolyPhen, among the earliest, are the most cited core VIPs (Fig. 3 and 4).

## Discussion and Conclusions

VIPdb version 2 provides a comprehensive view of VIPs. To identify the most appropriate VIPs for user’s specific needs, users are advised to thoroughly assess the strengths and weaknesses of VIPs before determining their suitability for use. For example, initiatives like the Critical Assessment of Genome Interpretation (CAGI) conduct community experiments to assess VIPs across different variant types and contexts (8, 9, 10).

With 403 curated VIPs, VIPdb version 2 provides a comprehensive overview of programs designed for variant impact prediction, along with their characteristics, citation patterns, publication details, and access information. VIPdb version 2 is available on the CAGI website (https://genomeinterpretation.org/vipdb). We invite submissions of new VIPs to the next version of VIPdb.

## Data Availability

The VIPdb database is available on the CAGI website (https://genomeinterpretation.org/vipdb), and the full version can be downloaded as a spreadsheet.

## List of abbreviations

VIP: Variant Impact Predictor
VIPdb: Variant Impact Predictor Database
